# Electrically Tunable Two-Color Cholesteric Laser

**DOI:** 10.3390/polym15244656

**Published:** 2023-12-09

**Authors:** Lotfi Saadaoui, Donghao Yang, Yu Wang, Faheem Hassan, Irena Drevensek-Olenik, Xinzheng Zhang, Zenghua Gan, Yigang Li, Jingjun Xu

**Affiliations:** 1 The MOE Key Laboratory of Weak-Light Nonlinear Photonics and International Sino-Slovenian Join Research Center on Liquid Crystal Photonics, TEDA Institute of Applied Physics and School of Physics, Nankai University, Tianjin 300457, China; lotfi.saadaoui@fst.utm.tn (L.S.); yangdonghao0305@126.com (D.Y.); 1120190080@mail.nankai.edu.cn (Y.W.); faheemhassan118@hotmail.com (F.H.); 2120210201@mail.nankai.edu.cn (Z.G.); liyigang@nankai.edu.cn (Y.L.); jjxu@nankai.edu.cn (J.X.); 2 Physics Laboratory of Soft Matter and Electromagnetic Modelling, Faculty of Sciences of Tunis, University of Tunis El Manar, Tunis 2092, Tunisia; 3 Faculty of Mathematics and Physics, University of Ljubljana, SI-1000 Ljubljana, Slovenia; 4 Department of Complex Matter, J. Stefan Institute, SI-1000 Ljubljana, Slovenia; 5 Collaborative Innovation Center of Extreme Optics, Shanxi University, Taiyuan 030006, China

**Keywords:** cholesteric liquid crystal, band edges, two-color lasing, organic fluorescent

## Abstract

Two-color lasing emission from an asymmetric structure, consisting of two dye-doped cholesteric liquid crystal (DD-CLC) layers separated by a transparent interlayer, is demonstrated. The DD-CLC mixtures have different reflection bands with long-wavelength band edges located at the green and red wavelengths of the visible spectrum, respectively. For the laser action, the CLC hosts provide the feedback, and the fluorescent laser dyes represent the active medium. When the stacked structure is optically pumped above the threshold, two simultaneous laser lines separated by 123 nm are observed at the long-wavelength band edges of the DD-CLC mixtures. The influence of an electric field on lasing behavior is also analyzed and discussed in terms of the reflection spectrum and laser action. The results show a reversible tuning of the reflection band, accompanied by a modification of the lasing characteristics under the application of an external field. Above a specific threshold voltage, one of the emission lines is suppressed and the other is conserved. With a further increase in the voltage, both laser emissions are entirely inhibited. The investigated structure demonstrates a simple technique to obtain an electrically tunable multi-wavelength laser, which might pave the way for a new generation of organic laser sources.

## 1. Introduction

Dye-doped cholesteric liquid crystal (DD-CLC) lasers have attracted significant interest due to their cheap and simple fabrication process and stable operation. Their compact design makes them easy to assemble, while their capability to emit different wavelengths by using a sole pumping beam makes them very convenient for the construction of tunable laser sources. The emitted laser frequency can be readily fine-tuned by manipulating various external or internal factors, further emphasizing their adaptability for diverse applications. CLCs are one-dimensional photonic crystals with a self-organized regular helical arrangement generating a periodic modulation of the refractive index [1]. The optical period of the structure corresponds to half of the helix pitch, which is defined as *p* = *λ*_0_/*n*, where *λ*_0_ is the central wavelength of the photonic band gap (PBG) and *n* is the average refractive index defined as <*n*> = (*n_e_* + *n_o_*)/ 2, where *n_e_* and *n_o_* are the extraordinary and ordinary indices of refraction, respectively. The intrinsic helical modulation of CLCs imparts them with numerous remarkable properties; for instance, it enables them to operate as a distributed feedback (DFB) assembly in CLC lasers [2]. This property of CLCs provides a very promising approach for creating compact laser sources with an inherent low threshold and stable emission [2,3]. To obtain the optimal lasing performance, the integration of a luminescent dopant into a pure CLC should be performed in such a way that its fluorescence spectrum matches with one of the CLCs’ photonic band edges [4,5]. The resulting band-edge lasers are distinguished by their stimulated emission, which is boosted at the edge of the PBG and inhibited within the PBG [6,7,8]. This is because, according to Fermi’s golden rule, the luminescence intensity is proportional to the density of photonic states [9,10], which is maximal at the band edges [11]. A significant advantage of CLC lasers lies in their capacity to adjust the emission wavelength by manipulating the CLC pitch, which can be achieved either by changing the temperature [12,13] or the composition [14] of the CLC material, by means of the photochemical manipulation of the constituent molecules via the photo-transformation effects of aromatic esters [15], azo and azoxy compounds [16], or via mechanical stretching in cross-linked CLC elastomers [17,18]. One of the very attractive control methods is tuning via an applied electric field; however, its full potential has not yet been achieved [19]. Recently, several approaches to achieve lasing at different wavelengths have been demonstrated using a CLC multilayer system [20], emulsified polymer-dispersed liquid crystals (PDLCs) [21], CLC spherical micro-shells [22], and a defect mode laser structure [23] and topological lasing [24]. For instance, Barberi et al. demonstrated multi-wavelength lasing through a combination of a photoluminescent dye layer and three cholesteric layers [20]. Multi-wavelength lasing from a polymer–cholesteric liquid crystal superstructure with coexisting opposite chiralities was also obtained by refilling a right-handed polymeric scaffold with a left-handed cholesteric liquid crystal material [23]. In addition, robust topological interface state lasing at multiple wavelengths of the visible spectrum in a micron-sized polymer-cholesteric liquid crystal superlattice was also demonstrated [24]. Nevertheless, the generated laser emissions only spanned a limited region of the visible spectrum, impacting their versatility and reducing their potential applications.

In this work, we present simultaneous dual-wavelength lasing from a DD-CLC system. A new cell configuration involving two DD-CLC layers with two different organic fluorescent laser dyes is proposed. Using this method, one can obtain simultaneous lasing emission at two distant wavelengths, in our case in the green and red regions of the visible spectrum. The laser lines are separated by 123 nm and are located at the long-wavelength band edges of the two CLC materials. Furthermore, we demonstrate that lasing characteristics can be modified by the application of an external field. Interestingly, when increasing the voltage, total suppression is observed for the first emission line (in the green region), only a decrease in the intensity is observed for the second line (in the red region). With a further increase in the electric voltage, both laser emissions are entirely suppressed.

## 2. Materials and Methods

### 2.1. Materials

Cholesteric liquid crystals were prepared by mixing the nematic liquid crystal QYTN-009 and the right-handed chiral dopant R5011 with a high helical twisting power of 116 μm^−1^ at 20 °C, both from Qingdao QY Liquid Crystal Co., Ltd., Qingdao, China. The extraordinary and ordinary refractive indices of QYTN-009 are *n*_e_= 1.675 and *n*_o_= 1.516 at T = 20 °C, respectively. As fluorescent dyes, a red dye, 4-(dicyanomethylene)-2-methyl-6- (4 dimethyl aminostyryl)-4H-pyran (DCM), and a green dye, (3-(2-benzothiazole)-7 (diethylamino) -2H-1-benzopyran-2-one) (C540A), both available from Exciton, Dayton, OH, USA, were used. A stacked cell composed of two dye-doped CLC mixtures was prepared by using three ITO-coated glass plates separated by 10 µm spacers. By virtue of this combination, a single UV pumping beam could be used to achieve simultaneous lasing at the red and green wavelengths where the long-wavelength band edges were placed [1,25]. In addition, the transition dipole moments of the DCM and C540A dyes held positive-order parameters when situated within the liquid crystal (LC) hosts [26,27]. This indicated that the orientation of these transition dipole moments was aligned with the local molecular alignment within the LC molecules, which gave rise to greater overlap between the transition dipole moment of dye molecules and the polarization vector of the incident electromagnetic wave [28]. As a result, preferential laser emission could occur at the long photonic band edge [1]. To obtain the planar orientation of the CLC molecules, the inner surfaces of the glass plates were coated with a layer of polyvinyl alcohol (PVA) (Sigma Aldrich) and then rubbed in the parallel direction. PVA was dissolved in water at a concentration of 0.5% in weight. The following mixtures were prepared and introduced in the first and the second layers:

(i) CLC1/DCM: 99% (97.6 wt. % QYTN-009 + 2.4 wt. % R5011) + 1 wt.% DCM
(ii) CLC2/C540A: 99% (96.9 wt. % QYTN-009 + 3.1 wt. % R5011) + 1 wt.% C540A

During the filling process, the mixtures were heated to a temperature above the clearing point to ensure uniform dispersion. After the preparation of the CLCs/dyes cell, the sample was cooled to room temperature.

### 2.2. Optical Excitation

**Figure 1:**
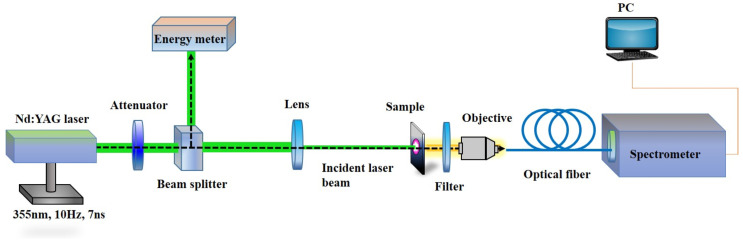
Experimental setup.

The experimental setup used in our study is described in Figure 1. The third harmonic radiation from a pulsed Q-switched Nd:YAG laser was used as a pumping light source (SLIII-10, Continuum, Dallas, TX, USA). The pulse wavelength, width, and repetition rate were 355 nm, 7 ns, and 10 Hz, respectively. The maximum pulse energy was about 170 µJ. The incident beam was used to pump the cell in the normal direction. The beam intensity was controlled by neutral density filters (attenuator). A beam splitter (BS) was used to split the incident light into the transmitted part, to stimulate the sample, and the reflected part, to detect the power via the energy meter. To focus the incident light on the cell to a spot size with diameter of tens of micrometers, a lens with a focal length of 10 cm was placed between the BS and the sample. The emitted light from the sample was collected by a 10× microscope objective and then transmitted via an optical fiber to a high-resolution spectrometer (SP2358, Princeton Instruments, Trenton, NJ, USA). To protect the spectrometer and reject the pump beam, a 355 nm notch blocking filter was placed between the sample and the objective. The cell was pumped from the side of the CLC_1_/DCM layer in order to avoid absorption of the emitted laser light in the second CLC layer, and all measurements were carried out under ambient conditions at 25 °C.

## 3. Results

**Figure 2:**
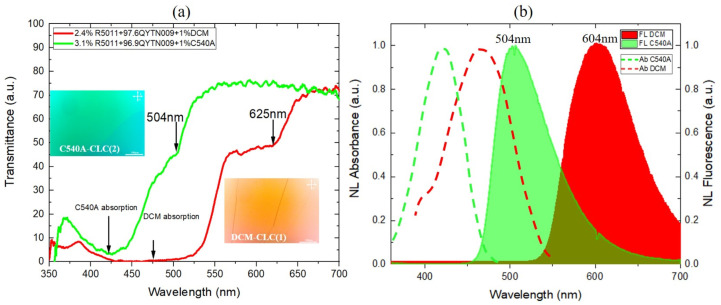
(**a**) The transmission spectra of the CLC_1_/DCM (red curve) and CLC_2_/C540A (green curve) mixtures. The insets show POM images that reveal textures of oily streaks in both mixtures. (**b**) The normalized (NL) absorption spectra (dash curves) and NL fluorescence spectra (solid curves) of the DCM (red curves) and C540A (green curves) dyes.

In order to better understand the basic mechanisms of double laser emission, the optical properties of the DD-CLC mixtures were first investigated separately. An HR4000CG-UV-NIR high-resolution spectrometer (Ocean Optics, Duiven, The Netherlands) coupled with a UV-Vis-NIR light source (DH-2000-BAL, Ocean Optics, Duiven, The Netherlands) was used to analyze the spectral response of the DD-CLC mixtures and their photonic band edges. Figure 2a shows the corresponding transmission spectra indicating their relative position in order to achieve lasing at the long-wavelength band edges using a sole pumping beam. The long-wavelength band edges are situated at the wavelengths of 504 nm and 625 nm, respectively. The inset pictures show the polarization optical microscopy (POM) images of the two mixtures inserted into two 10 μm thick cells. The oily streak texture approves the planar alignment of both mixtures.

The long-wavelength band edges for both mixtures are chemically controlled by changing the chiral dopant concentration, in order to align with the maximum peak of the dye fluorescence spectrum and to generate simultaneous laser lines in the red and green wavelength of the visible spectrum. In Figure 2b, the absorption (dashed lines) and fluorescence (solid lines) spectra of the DCM and C540A laser dyes measured in the nematic host used in this study are presented. As illustrated, the absorption spectra of both layers cover a significant region of the visible spectrum, presenting a substantial opportunity for effective optical excitation with the third harmonic Nd:YAG laser. Specifically, the first DCM dye in the nematic host exhibits a wide absorption band ranging from the UV region to 550 nm and provides efficient fluorescence in the visible spectrum (precisely between ~550 and 700 nm with a full width at half maximum (FWHM) of ~89 nm), with a distinguished maximum at 604 nm. To achieve emission in the red region of the visible spectrum, the long-wavelength band edge of the first mixture was fixed to cover the red portion of the spectrum with respect to the fluorescence efficiency of the DCM dye. The second C540A/nematic mixture also exhibits absorbance in the UV region up to the visible region (≈350–500 nm) and provides significant fluorescence in the green region (≈450–620 nm with an FWHM of ~74.1 nm), with a maximum around 504 nm.

**Figure 3:**
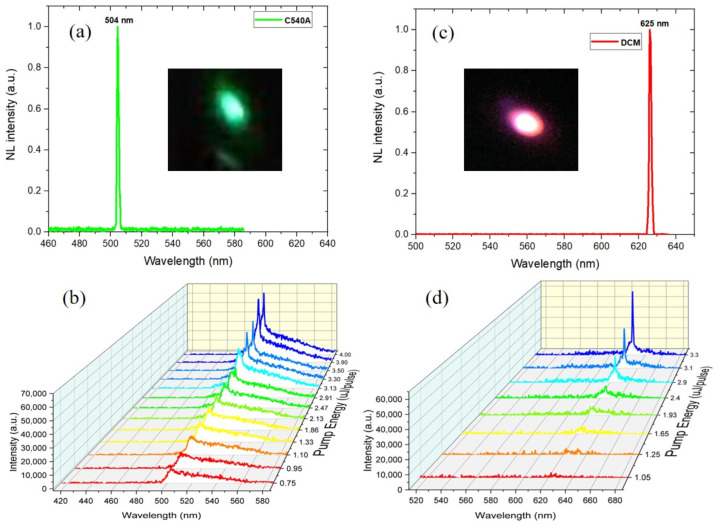
Lasing emission spectra of two separate individual cells: (**a**) CLC_2_/C540A cell and (**c**) CLC_1_/DCM cell (at pump energy of ~5 μJ/pulse). The insets show the two laser beams in far field. Emission spectra of the two cells versus the pump energy: (**b**) CLC_2_/C540A cell and (**d**) CLC_1_/DCM cell.

Initially, the lasing characteristics of two single cells were analyzed. With this purpose in mind, two 10 μm thick commercial cells (Instec, Inc., Boulder, CO, USA) were used. Figure 3a,b show the laser emissions from the CLC_1_/DCM and CLC_2_/C540A cells, respectively. Both cells are found to emit monochromatic laser lines at 504 nm (green) and 625 nm (red), with FWHMs of 1.51 nm and 1.39 nm, respectively. The pumping energy was around 5 µJ/pulse for the two cells. The two insets show the far-field images of the output beams. The output emission intensity versus the pump energy of the excitation laser pulse was also studied. The corresponding emission spectra are presented in Figure 3b,d.

Commonly, laser dyes have the capability to emit across a wide wavelength range. Since the DCM and C540A dyes align well with the local director of the CLC and the density of photonic states at the low-energy band edge is higher than that at the high-energy edge, laser lines occurred at the long-wavelength band edges. For the DCM dye, the laser line has a 20 nm red shift from its fluorescent peak, which provides a significant level of tunability for band edge lasers.

**Figure 4:**
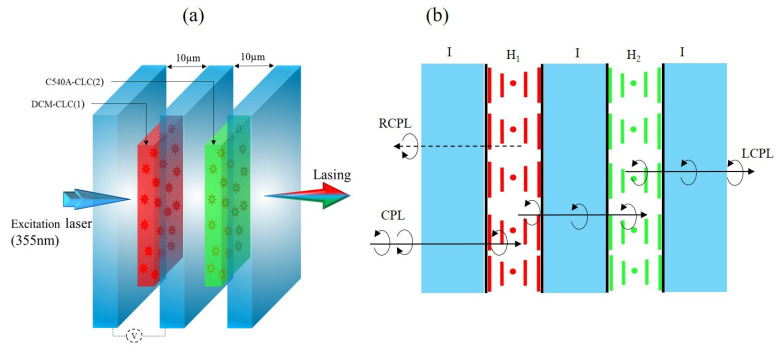
(**a**) Sketch of the multi-layer structure. (**b**) Schematic illustration of the system: I: isotropic layer; H_i_ (i = 1, 2): CLC_1_/DCM and CLC_2_/C540A layers.

In order to achieve a dual-wavelength laser, a new cell configuration was prepared by stacking the two layers (a CLC_1_/DCM layer and a CLC_2_/C540A layer) one on the top of the other with a separation glass plate positioned in between them. A sketch of the stacked cell is depicted in Figure 4a.

When an incident beam pumped the stacked structure in the normal direction, two simultaneous laser lines were observed. The first line occurred at 504 nm and the second was observed at 627 nm, and their FWHMs were 1.76 nm and 1.15 nm, respectively. With respect to the single cell, the red peak had a red shift of 2 nm and a narrower FWHM. The alignment layers of the glass cell have the capability to enforce a strict restriction of the helix to a half integer of the pitch. This means that a small variation in the cell thickness can cause a slight change in the helix pitch at different locations of the cell [29]. As a result, a fluctuation (~3 nm) of the laser peak wavelength can occur. The pumping energy was around 7.6 μJ/pulse.

**Figure 5:**
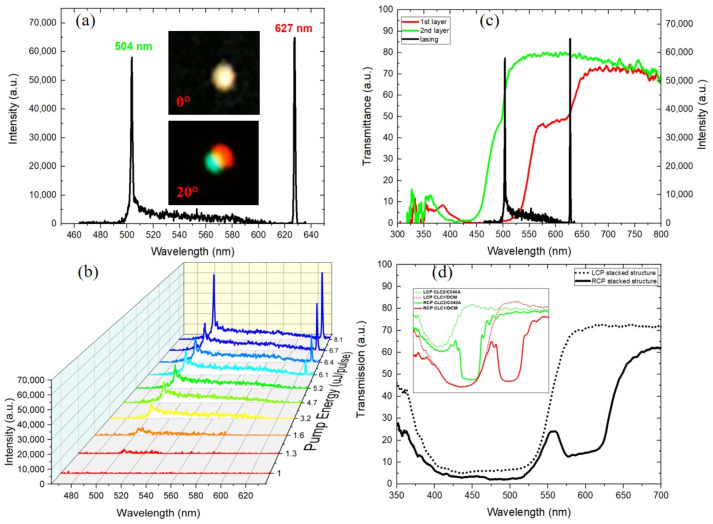
(**a**) Dual-wavelength lasing spectrum with emission lines positioned at 504 nm and 627 nm detected under 7.6 μJ/pulse excitation energy. Inset: Images of the output beam by pumping the cell at an angle of 0° and 20° relative to the cell normal. (**b**) Emission spectrum vs. pump energy. (**c**) Lasing spectrum of multi-layer cell and transmission spectra of both mixtures. (**d**) Transmission spectra for RCP and LCP incident light of the stacked cell. Inset: The transmission spectra of individual cells.

In Figure 5a, a photograph of the two laser lines emitted from the cell is shown. It is evident from Figure 5c that the emitted laser lines correspond to the long-wavelength band edges of the PBGs. The insets depict the output beams obtained by pumping the cell at an angle of 0° and for an oblique incidence relative to the cell normal. At an oblique incidence, the long-wavelength band edge of the cholesteric mixtures experienced a blue shift by ≈10 nm at an incidence of 20°, with good agreement with [30]. The emitted laser spots were captured roughly 5 cm away from the cell. The laser peaks are separated by about 123 nm. In general, this distance can be controlled by adjusting the long-wavelength band edge of the CLCs and the fluorescence and absorption spectra of laser dyes. The investigated dual-layer combination allows the generation of lasing emission spanning from the ultraviolet (UV) to the infrared (IR) regions of the electromagnetic spectrum, thus expanding their potential use in a variety of fields, notably in optical communications, sensors and biosensors devices. In addition, Figure 5b shows the variation of the lasing output intensity with the pumping energy. The lasing threshold of the dual-layered system is about 8.1 μJ/pulse, which is about two times larger than in the single-cell samples. The increase in the lasing threshold can be explained by the multiple internal reflections and the radiation loss produced at the interfaces [31].

The propagation of light and the lasing characteristic of the structure investigated in our study are sketched in Figure 4b and can be described as follows: the incident light at 355 nm entering the bilayer helical structure is decomposed into right (R) and left (L) circularly polarized components. The circularly polarized light (CPL) with the same handedness as the cholesteric helix is reflected, while the CPL with the opposite handedness is transmitted through the system and excites the dyes.

In the investigated system, due to the right-handedness of the helical structure, the RCPL is reflected and LCPL is transmitted through the first layer. Figure 5d exhibits the transmission spectra for RCP (solid line) and LCP (dashed line) incident light of the stacked cell (black curve) and individual cells (green and red curves in the inset). Passing through the first layer, the light stimulates the gain medium by exciting the molecules of the DCM dye, by which the stimulated emission process occurs. This process takes place inside the helical structure that provides the distributed feedback for lasing action. Then, the transmitted light passes through the glass plate with preserved polarization. Afterwards, the slightly attenuated pump light enters the second layer with the same helicity (LCPL) [32,33], and consequently induces stimulated emission in the second active medium (C540A dye). In principle, lasing emission is possible for both CPL components, but only the LCPL can reach a strong enough gain. No stimulated emission is detected at low pump energies. By continuously increasing the excitation energy, at first, the second layer (CLC_2_/C540A) emits a laser line at 504 nm (green). At an even higher pumping energy, the second line from the CLC_1_/DCM layer is detected simultaneously with the first line (see Figure 5c). To avoid absorption of the emission from the CLC_2_/C540A layer in the CLC_1_/DCM layer, the pump beam was incident on the structure from the side of the CLC_1_/DCM layer. The structure demonstrated in this work presents a straightforward preparation approach achieved by minimizing the overall number of cholesteric layers while integrating both the feedback from CLC and the stimulated emission from the laser dye in each layer.

**Figure 6:**
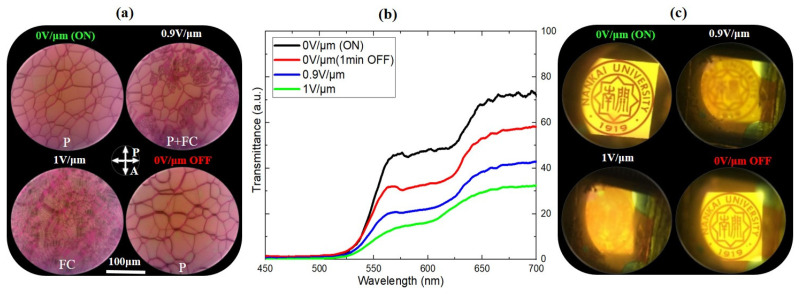
(**a**) POM images, (**b**) transmission spectra and (**c**) transparency for white light at different applied voltages. P: planar; FC: focal conic states.

Liquid crystalline molecules exhibit exceptionally strong susceptibility to the presence of an external electric field [34,35,36]. In the absence of an external field, molecular orientation is influenced by the surface alignment layers. The liquid crystal used in our study has a positive dielectric anisotropy, being approximately 9.6 at 20 °C. Consequently, the application of the external field tends to reorient the LC molecules in the direction of the field and has the capability to adjust the emission from the stacked cell. To study this effect, an electric field was applied across the first layer (DCM/CLC1) using a square wave-signal (1 kHz) from a function generator (Agilent-33120A, Boulder, CO, USA) via the ITO conductive layers. The application of an external field distorted the cholesteric texture and decreased the cholesteric order in the exposed layer. As a result, the PBG of the CLC_1_/DCM mixture exhibited a distortion when increasing the external voltage, as depicted in Figure 6a–d.

**Figure 7:**
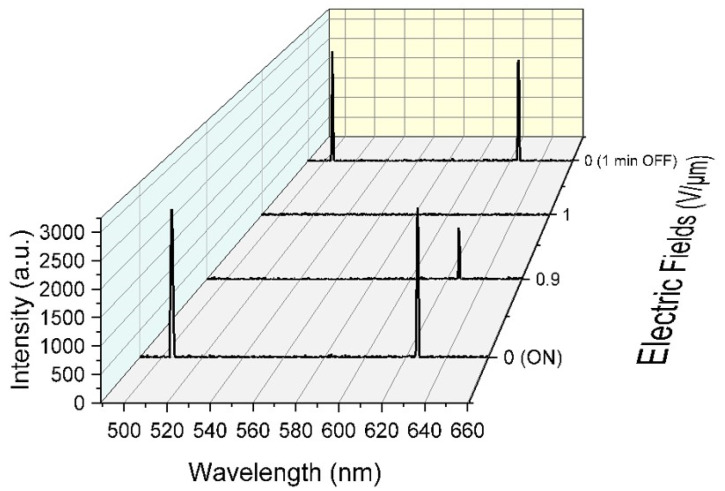
Lasing emission at different voltages (0V/μm, 0.9V/μm, and 1V/μm) and 1 min after the removal of the applied voltage.

At zero voltage, the characteristic oily streak texture of planar alignment was observed, as shown in Figure 6a [37]. In this texture, the helical axis was aligned perpendicularly to the cell surfaces and the long-wavelength band edge of the PBG was located at 625 nm. The cell transmittance for white light was more than 70%, as presented in Figure 6c. The lasing emission spectra at different external electric fields are displayed in Figure 7. When the electric field of E ≈ 0.9 V/μm was applied, a focal conic (FC) texture started to spread on the active surface [37,38,39]. This state was characterized by partial light scattering accompanied by a decrease in the transmittance (~ 40%, blue curve in Figure 6b) due the disturbance of the helical structure and the partial distortion of the PBG (Figure 6b). As a result, the pump beam was perturbed and consequently the green emission line vanished, while the intensity of the red emission line was decreased (Figure 7). At E = 1 V/μm, the Grandjean planar texture collapsed into the FC texture and the whole active surface was covered by the FC state, as can be seen in Figure 6a. Strong light scattering was produced (only 30% of the incident light could cross the sample) (Figure 6c), accompanied by the disorder of the helical structure and distortion of the PBG, as shown in Figure 6b [40]. As a result, no laser emission was produced in this case (Figure 7).

After removing the applied voltage, the helical structure was restored, which resulted in the restoration of the PBG with the long-wavelength band edge at the same wavelength as before (Figure 7). The recovery of the cholesteric order was accompanied by the restoration of the laser emission at both wavelengths. Two simultaneous laser lines were observed 1 min after switching off the voltage, as seen in Figure 7.

## 4. Conclusions

We introduce a novel assembling approach to achieve dual-wavelength lasing through the combination of two cholesteric materials and two organic dyes forming a bi-layer system. This new cell configuration yielded two distinct laser lines, separated by 123 nm, positioned at the long-wavelength band edges of the cholesteric materials. Notably, these wavelengths fell within the green and red regions of the visible spectrum, indicating the potential for developing a new generation of organic laser sources. In addition, we showcase the remarkable ability to dynamically modulate the intensities of these dual two laser lines by the application of an external voltage. The presented results show that beyond a threshold voltage, one of the laser lines is suppressed, while the other remains preserved. As the voltage is further increased, both laser emissions are completely restrained. This method could be an attractive way for building low-intensity broadband laser sources, with promising applications in optical devices such as tunable lasers and reflective displays.

## Data Availability

The data that support the findings of this study are available from the corresponding author upon reasonable request.

## References

[1] Yablonovitch E. (1987). Inhibited spontaneous emission in solid-state physics and electronics. Phys. Rev. Lett..

[2] Ortega J., Folcia C.L., Etxebarria J. (2017). Upgrading the performance of cholesteric liquid crystal lasers: Improvement margins and limitations. Materials.

[3] Ilchishin I.P., Tikhonov E.A. (2015). Dye-doped cholesteric lasers: Distributed feedback and photonic bandgap lasing models. PQE.

[4] Uchimura M., Watanabe Y., Araoka F., Watanabe J., Takezoe H., Konishi G. (2010). Development of laser dyes to realize low threshold in dye-doped cholesteric liquid crystal lasers. Adv. Mater..

[5] Wang N., Evans J.S., Mei J., Zhang J., Khoo I.-C., He S. (2015). Lasing properties of a cholesteric liquid crystal containing aggregation-induced-emission material. Opt. Express.

[6] Dolganov P.V., Dolganov V.K. (2018). Photon density of states in a cholesteric photonic crystal. JETP Lett..

[7] Dolganov P. (2015). Density of photonic states in cholesteric liquid crystals. Phys. Rev. E.

[8] Yablonovitch E. (1993). Photonic band-gap structures. J. Opt. Soc. Am. B.

[9] Gao S., Zhai Y., Zhang X., Song X., Wang J., Drevensek-Olenik I., Rupp R.A., Xu J. (2018). Coupling of Defect Modes in Cholesteric Liquid Crystals Separated by Isotropic Polymeric Layers. Polymers.

[10] Mavrogordatos T.K., Morris S., Castles F., Hands P., Ford A., Coles H., Wilkinson T. (2012). Density of photon states in dye-doped chiral nematic liquid crystal cells in the presence of losses and gain. Phys. Rev. E.

[11] Gevorgyan A., Oganesyan K., Karapetyan R., Rafayelyan M. (2013). The photonic density of states and the light energy density in cholesteric liquid crystal cells. Laser Phys. Lett..

[12] Lee S.S., Kim J.B., Kim Y.H., Kim S.-H. (2018). Wavelength-tunable and shape-reconfigurable photonic capsule resonators containing cholesteric liquid crystals. Sci. Adv..

[13] Humar M., Muševič I. (2010). 3D microlasers from self-assembled cholesteric liquid-crystal microdroplets. Opt. Express.

[14] Eelkema R., Pollard M.M., Katsonis N., Vicario J., Broer D.J., Feringa B.L. (2006). Rotational reorganization of doped cholesteric liquid crystalline films. J. Am. Chem. Soc..

[15] Petriashvili G., Matranga M., De Santo M.P., Chilaya G., Barberi R. (2009). Wide band gap materials as a new tuning strategy for dye doped cholesteric liquid crystals laser. Opt. Express.

[16] Chilaya G., Chanishvili A., Petriashvili G., Barberi R., Bartolino R., De Santo M.P., Matranga M., Collings P. (2006). Light control of cholesteric liquid crystals using azoxy-based host materials. Mol. Cryst. Liq. Cryst..

[17] Zhang P., de Haan L.T., Debije M.G., Schenning A.P. (2022). Liquid crystal-based structural color actuators. Light Sci. Appl..

[18] Varanytsia A., Nagai H., Urayama K., Palffy-Muhoray P. (2015). Tunable lasing in cholesteric liquid crystal elastomers with accurate measurements of strain. Sci. Rep..

[19] Ryabchun A., Bobrovsky A. (2018). Cholesteric liquid crystal materials for tunable diffractive optics. Adv. Opt. Mater..

[20] Saadaoui L., Petriashvili G., De Santo M.P., Hamdi R., Othman T., Barberi R. (2015). Electrically controllable multicolor cholesteric laser. Opt. Express.

[21] Petriashvili G., De Santo M.P., Hernandez R.J., Barberi R., Cipparrone G. (2017). Mixed emulsion of liquid crystal microresonators: Towards white laser systems. Soft Matter.

[22] Chen L., Li Y., Fan J., Bisoyi H.K., Weitz D.A., Li Q. (2014). Photoresponsive monodisperse cholesteric liquid crystalline microshells for tunable omnidirectional lasing enabled by a visible light-driven chiral molecular switch. Adv. Opt. Mater..

[23] Yang D., Chemingui M., Wang Y., Zhang X., Drevensek-Olenik I., Hassan F., Wu Q., Li Y., Saadaoui L., Xu J. (2023). Dual-wavelength lasing with orthogonal circular polarizations generated in a single layer of a polymer–cholesteric liquid crystal superstructure. Polymers.

[24] Wang Y., Yang D., Gao S., Zhang X., Drevensek-Olenik I., Wu Q., Chemingui M., Chen Z., Xu J. (2023). Tunable Topological Lasing of Circularly Polarized Light in a Soft-Matter-Based Superlattice. Laser Photonics Rev..

[25] Kopp V., Genack A. Lasing at the edge of a photonic stop band in cholesteric liquid crystals. Proceedings of the IEEE LEOS.

[26] Shirvani-Mahdavi H., Ebrahimi-Azandariani S. (2019). Effect of combined laser dyes on the efficiency of cholesteric liquid crystal lasers. JTAP.

[27] Schmidtke J., Stille W., Finkelmann H., Kim S.T. (2002). Laser emission in a dye doped cholesteric polymer network. Adv. Mater..

[28] Schmidtke J., Stille W. (2003). Fluorescence of a dye-doped cholesteric liquid crystal film in the region of the stop band: Theory and experiment. Eur. Phys. J. B.

[29] Morris S.M., Ford A.D., Broughton B.J., Pivnenko M.N., Coles H.J. (2005). Liquid crystal lasers: Coherent and incoherent microsources. Proceedings of the Emerging Liquid Crystal Technologies.

[30] Ozaki R. (2019). Simple model for estimating band edge wavelengths of selective reflection from cholesteric liquid crystals for oblique incidence. Phys. Rev. E.

[31] Mitov M. (2012). Cholesteric liquid crystals with a broad light reflection band. Adv. Mater..

[32] Gevorgyan A., Oganesyan K., Vardanyan G., Matinyan G. (2014). Photonic density of states of cholesteric liquid crystal cells. Laser Phys..

[33] Gevorgyan A., Harutyunyan M., Matinyan G., Mkhitaryan S. (2014). Photonic density of states of a stack of cholesteric liquid crystal and isotropic medium layers. Proceedings of the Journal of Physics: Conference Series.

[34] Adamow A., Szukalski A., Sznitko L., Persano L., Pisignano D., Camposeo A., Mysliwiec J. (2020). Electrically controlled white laser emission through liquid crystal/polymer multiphases. Light Sci. Appl..

[35] Saadaoui L., Hamdi R.J. (2021). Electrically tunable cholesteric liquid crystal lines defects. Opt. Mater..

[36] Meyer R.B. (1968). Effects of electric and magnetic fields on the structure of cholesteric liquid crystals. Appl. Phys. Lett..

[37] Lotfi S., Hamdi R., Othman T. (2018). Optical properties of frustrated cholesteric liquid crystals in planar cell. Phase Transit..

[38] Chilaya G. (2001). Cholesteric liquid crystals: Optics, electro-optics, and photo-optics. Chirality in Liquid Crystals.

[39] Dolganov P., Baklanova K., Dolganov V. (2022). Peculiarities of focal conic structure formed near the cholesteric-isotropic phase transition. Phys. Rev. E.

[40] Cheng W.-F., Lai J.-C., Jie S.T.F., Liu C.-K., Cheng K.-T. (2019). Scattering-/absorption-mode light shutters based on dye-doped fingerprint chiral textures. Dye. Pigm..

